# Importance of halide involving interactions at Hoogsteen sites in supramolecular architectures of some coordination metal complexes of N^6^-benzyl/furfuryl adenine

**DOI:** 10.1186/s13065-014-0058-z

**Published:** 2014-10-29

**Authors:** Samson Jegan Jennifer, Packianathan Thomas Muthiah, Duraiswamy Tamilselvi

**Affiliations:** School of Chemistry, Tiruchirappalli-620024, Tamil Nadu, India

**Keywords:** N^6^-benzyladenine, N^6^-furfuryladenine, X-ray diffraction studies, Crystal structures, Supramolecular architectures

## Abstract

**Background:**

Most of the benzyladenine and furfuryladenine derivatives inhibit tumor/cancer cell growth; their toxicity is lesser than the compounds used for the treatment of cancer now-a-days. Many cytokinin derivatives are tested for anticancer activity.

**Results:**

A series of transition metal complexes containing N^6^-benzyl/furfuryl aminopurines of formula [Mn(FAH)_2_(H_2_O)(Cl_3_)]_2_.Cl_2_**(1),** [Co(FAH)2(H_2_O)(Cl_3_)]_2_.Cl_2_**(2),** [Co(FAH)_2_(Cl_4_)]_2 ._[Co(FAH)_2_(H_3_O)(Cl_3_)].Cl_2_**(3),** [Ni(FAH)_2_(H_2_O)(Cl_3_)]_2_.Cl_2._ (H2O) **(4),** [Zn(BAH)Br_3_] **(5)** and [Cd_2_(BAH)_2_(μ-Br)_4_Br_2_]_n_**(6)** (where BAH and FAH benzyladeninium and furfuryladeninium cations respectively**)** have been synthesized and characterized. Crystal structures of **(1-4)** have similar distorted octahedral coordination geometry, while **(5)** and **(6)** have distorted tetrahedral geometry and octahedral geometries respectively. In **(1-4)** two halide ions and two cytokinin cations (BAH^+^/FAH^+^) are laterally coordinated to the metal ion. A water molecule and a halide ion are axially coordinated. But the coordination sphere of **(5)** consists of N7 coordinated benzyladeninium ion and three halide ions. The complex (**6**) is a coordination polymer bridged by bromide anions. A common notable feature in **(1-4)** is the presence of one or more lattice chloride anions. They help in a chain formation by N-H…Cl halide involving hydrogen bonding interactions in between the Hoogsteen site hydrogen.

**Conclusions:**

The observed crystal structures emphasize the role of the halide ions in developing the supramolecular architectures by halide involving hydrogen bonding interactions. Also most of the reported cobalt cytokinin complexes possess tetrahedral coordination geometry, but some cobalt complexes have distorted octahedral coordination geometry, which are discussed and compared.

**Graphical Abstract:**
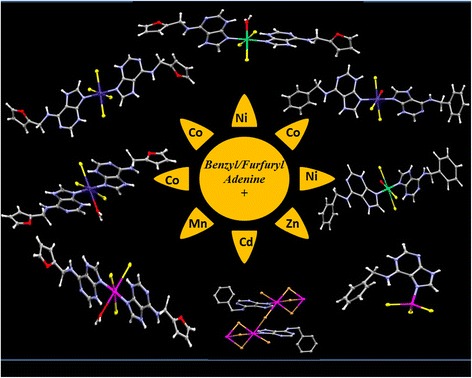
Supramolecular architectures of some coordination metal complexes of N6-benzyl/furfuryl adenine.

## Background

Molecular crystal engineering involving an organic ligand coordinated to metal ions which are self assembled in the formation of supramolecular architectures is a field of evergreen interest. This self assembly process is governed by a variety of hydrogen bonding and π-π stacking interactions. These interactions play a vital role in controlling the properties and in turn the potential applications of the organic-inorganic hybrid materials [1]. Their applications involve a wide spectrum ranging from molecular sensing ion-exchange catalysis, magnetism, and gas storage [2-6]. The transition metals such as cobalt, zinc, iron, copper, platinum and palladium enhance the cytotoxic activity of the cytokinins on tumour cells [7-16].

Coordination site of the cytokinins is governed by various factors such as pH, nature of the metal, substituents on the aminopurine skeleton, etc [17-20]. Copper and cadmium cytokinin crystal structures were reported in our laboratory [21-23]. Some of the reported cobalt cytokinin complexes have very good anticancer activity (in vitro condition) than the derivatives tested for clinical trials [24].

### Experimental section

#### Materials and methods

All reagents, solvents and ligands used for syntheses were purchased from commercial sources and used as received.

#### Preparation of bis[bis(furfuryladeninN1-H ium) manganese(II) (mono aqua) (trichlorido)] chloride (FAMNCL) (1)

40 ml aqueous solution of MnCl_2_.4H_2_O (0.0495 g) was heated at 95°C for 30 min. over a water bath. 0.0717 g of furfuryladenine and 3 drops of conc. HCl were added to this solution, and the heating was continued for 90 min. The resultant solution was allowed to slow evaporation at room temperature. After a few days pale green crystals of **(1)** were obtained.

#### Preparation of bis[bis(furfuryladeninN1-H ium) cobalt(II) (mono aqua) (trichlorido)] chloride (FACOCLI) (2)

The preparation procedure of (**2)** was same as that of **(1)** except CoCl_2_.6H_2_O (0.0595 g) was used instead of MnCl_2_.4H_2_O. After a few days pink prismatic crystal of **(2)** were obtained.

#### Preparation of bis[bis(furfuryladeninN1-H ium) cobalt(II) (tetrachlorido)] [bis(furfuryladeninN1-H ium) cobalt(II) (mono aqua) (trichlorido)] chloride (FACOCLII) (3)

20 ml aqueous solution of CoCl_2_.6H_2_O (0.0538 g) was stirred at 40°C for 30 min. To this15 ml ethanolic solution of furfuyladenine (0.0717 g) and 0.0398 g of 3-pyridine sulfonic acid were added. It resulted in white turbidity, which was removed by the addition of 5 ml dil.HCl. The solution was further heated at 50°C for 90 min. The resultant solution was allowed to slow evaporation at room temperature. After a few days pink coloured precipitate was formed. The pink precipitate was recrystallized using methanol.

#### Preparation of [bis(furfuryladeninN1-H ium)nickel (II)(mono aqua) (trichlorido)] chloride monohydrate. (FANICL) (4)

The preparation procedure of **(4)** was same as that of 1 except of NiCl_2_.6H_2_O (0.0594 g) was used instead of MnCl_2_.4H_2_O. After a few days green prismatic crystal of **(2)** were obtained.

#### Preparation of zinc (II)benzyladeninN1-H ium tri bromido [Zn(BAH+)Br3] BAZNBR (5)

40 ml aqueous solution of ZnBr_2_ (0.0563 g) was heated at 70°C for 30 min. over a magnetic stirrer. 0.0563 g of benzyladenine and 4 drops of conc. HBr were added to this solution and heated to 60 min. The resultant solution was allowed to slow evaporation at room temperature. After a few days pale green crystals of **(5)** were obtained.

#### Preparation of catena-Poly [μ-bromido bis(benzyladeninN1-H ium) di cadmium (II) tri -μ-bromido] (BACDBR) (6)

40 ml aqueous solution of CdBr_2_.4H_2_O (0.0861 g) was heated at 55°C for 30 min. over a magnetic stirrer. 0.0563 g of benzyladenine and 4 drops of conc. HBr were added to this solution and heated to 60 min. The resultant solution was allowed to slow evaporation at room temperature. After a few days pale green crystals of **(6)** were obtained .

### Crystal structure determination

Intensity data sets were collected at room temperature, on a BRUKER SMART APEXII CCD [25] area-detector diffractometer equipped with graphite monochromated Mo Kα radiation (λ = 0.71073 Å). The data were reduced by using the program SAINT [25] and empirical absorption corrections were done by using the SADABS [25]. The structures were solved by direct methods using SHELXS-97 [26] and subsequent Fourier analyses, refined anisotropically by full-matrix least-squares method using SHELXL-97 [26] within the WINGX suite of software, based on F2 with all reflections. All carbon hydrogens were positioned geometrically and refined by a riding model with Uiso 1.2 times that of attached atoms. All non H atoms were refined anisotropically. The molecular structures were drawn using the ORTEP-III [27] and MERCURY [28]. Crystal data and the selected parameters for compounds **(1-6)** were summarized in (Tables 1 and 2) respectively. The crystals remained stable throughout the data collection.

**Table 1:** **Crystal data and structure refinement information for compounds (1-6)**

	**1**	**2**	**3**	**4**	**5**	**6**
Empirical Formula	C_20_H_22_Cl_3_MnN_10_ O_3_, Cl	C20 H22 Cl3 Co N10 O3, Cl	C20 H23 Cl3 Co N10 O3, C20 H20 Cl4 Co N10 O2, 2(Cl)	C20 H22 Cl3 N10 Ni O3, H2 O, Cl	C12 H12 Br3 N5 Zn	C12 H12 Br3 Cd N5
Formula weight	647.22	651.21	1320.85	668.98	531.36	578.38
Temp, K	296(2)	296(2)	296(2)	296(2)	296(2)	296(2)
λ (Å)	0.71073	0.71073	0.71073	0.71073	0.71073	0.71073
Crystal system	Monoclinic	Monoclinic	Triclinic	Orthorhombic	Monoclinic	Tetragonal
Space group	C2/c	C2/c	P-1	P2_1_2_1_2_1_	P2_1_/c	P4322
a (Å)	22.9002(2)	22.9118(10)	6.9750(1)	12.1423(8)	17.8589(3)	8.2348(3)
b (Å)	16.0257(2)	15.8863(10)	13.2227(2)	28.3096(14)	6.2237(1)	8.2348(3)
c (Å)	29.4693(3)	29.2987(14)	28.8861(4)	7.9306(4)	15.4551(3)	47.438(2)
α(°)	90	90	82.206(1)	90	90	90
β (°)	102.281(1)	102.659(4)	86.755(1)	90	106.310(1)	90
γ (°)	90	90	81.045(1)	90	90	90
V (Å3)	10567.5(2)	10405.0(10)	2605.62(7)	2726.1(3)	1648.68(5)	3216.9(2)
Z	16	16	2	4	4	8
ρ calcd (g/cm3)	1.627	1.663	1.684	1.630	2.141	2.388
μ (mm-1)	0.949	1.116	1.164	1.152	8.759	8.809
F(000)	5264	5296	1340	1368	1016	2176
Goodness-of-fit on F2	1.01	1.06	1.06	1.03	1.09	1.26
Final R1 index [I > 2σ(I)]	0.0444	0.0548	0.0567	0.0412	0.0331	0.0558
wR2 (all data)	0.1435	0.1825	0.1790	0.1038	0.0952	0.1147
Largest difference in peak and hole (e Å-3)	-0.34, 0.70	-0.66, 0.67	-1.44, 0.69	-0.32, 0.79	-1.02, 0.77	-0.95, 0.74

**Table 2:** **Selected bond parameters in 1-6**

**COMPLEX**	**D-H…A**	**D - H (Å)**	**H…A (Å)**	**D…A (Å)**	**D - H…A (°)**	**Symmetry operation**
1	N(6B)‒H(6B)…Cl(7)	0.86	2.49	3.337(3)	169	x,1-y,1/2 + z
	N(6C)‒H(6C)…Cl(9)	0.86	2.52	3.372(3)	169	
	N(6D)‒H(6D)…Cl(7)	0.86	2.52	3.366(3)	168	
	N(7B)‒H(7B)…Cl(7)	0.86	2.25	3.075(3)	161	x,1-y,1/2 + z
	N(7C)‒H(7C)…Cl(9)	0.86	2.29	3.120(3)	161	
	N(7D)‒H(7D)…Cl(7)	0.86	2.24	3.067(3)	161	
2	N(6B)‒H(6B)…Cl(9)	0.86	2.48	3.330(3)	169	
	N(6C)‒H(6C)…Cl(9)	0.86	2.51	3.358(3)	168	x,1-y,-1/2 + z
	N(6D)‒H(6D)…Cl(7)	0.86	2.50	3.351(3)	169	1/2 + x,-1/2 + y,z
	N(7B)‒H(7B)…Cl(9)	0.86	2.26	3.087(3)	161	
	N(7C)‒H(7C)…Cl(9)	0.86	2.26	3.086(3)	161	x,1-y,-1/2 + z
	N(7D)‒H(7D)…Cl(7)	0.86	2.32	3.137(3)	159	1/2 + x,-1/2 + y,z
3	N(6A)‒H(6A)…Cl(2)	0.86	2.50	3.345(5)	168	
	N(6B)‒H(6B)…Cl(1)	0.86	2.50	3.346(4)	169	
	N(6C)‒H(6C)…Cl(1)	0.86	2.51	3.352(5)	169	
	N(6D)‒H(6D)…Cl(2)	0.86	2.48	3.328(5)	169	1-x,-y,1-z
	N(7A)‒H(7A)…Cl(2)	0.86	2.24	3.064(5)	161	
	N(7B)‒H(7B)…Cl(1)	0.86	2.29	3.113(5)	160	
	N(7C)‒H(7C)…Cl(1)	0.86	2.29	3.116(5)	160	
	N(7D)‒H(7D)…Cl(2)	0.86	2.24	3.066(5)	161	1-x,-y,1-z
4	N(6A)‒H(6A)…Cl(4)	0.86	2.46	3.304(5)	167	-1 + x,y,z
	N(6B)‒H(6B)…Cl(5)	0.86	2.52	3.368(5)	167	-1 + x,y,z
	N(7A)‒H(7A)…Cl(4)	0.86	2.27	3.079(4)	157	-1 + x,y,z
	N(7B)‒H(7B)…Cl(5)	0.86	2.23	3.055(4)	161	-1 + x,y,z
5	N(1) ‒H(1)…Br(3)	0.86	2.50	3.238(2)	145	x,3/2-y,-1/2 + z
	N(9) ‒H(9)…N(3)	0.86	2.09	2.898(4)	156	-x,2-y,-z
6	N(6) ‒H(6)…Br(2)	0.86	2.85	3.683(8)	165	x,1 + y,z
	N(7) ‒H(7)…Br(2)	0.86	2.64	3.451(8)	158	x,1 + y,z

## Results and discussion

Single crystal x-ray diffraction studies reveal that **(1)** and **(2)** are isomorphous structures. The cytokinins in complexes **(1-4)**, are N3 protonated, N9 coordinated and N7-H tautomer, but in **(5)** it is N1-protonated, N7 coordinated and N9-H tautomer. This can be confirmed from the bond angle difference between the N9-H neutral kinetin, benzyladenine structures and their protonated compounds [29,30]. Axially coordinated adenine ligand moieties are arranged in a trans manner with respect to the central axis (passing through the N9 atoms and metal) in the compounds **(1-4)**. Also the dihedral angle between the adenine ring and the N6-substituent aromatic ring shows the compounds all are active cytokinins [31].

**Scheme 1:**
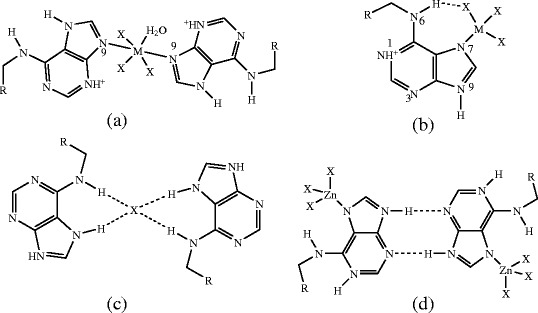
**In (a-d) X = Cl-,Br-.** (a-c) R = C_6_H_5_ for BA/C_4_H_3_O for FA. (d) R = C_6_H_5_ for BA.

A lattice halide ion bridges N6-H and N7-H (Hoogsteen site hydrogens) [32,33] via. N-H…X hydrogen bonds [shown schematically in Scheme 1c] which lead to a chain like pattern in the crystals **(1-4)**. A centrosymmetric base pair is formed due to the pairing of N3 and N9-H hydrogen bonds in (**5)** (Scheme 1d). Coordination spheres are arranged as a ladder by the coordinated chloride ion and water in structures **(1-3)** (only Co1 of 3). Ladders of **(1-3)** have a similar type of hydrogen bonds and atomic arrangements. There is no such ladder type pattern observed in Co2 and Co3 cobalt centers of **(3)** due to the absence of water molecule in the coordination sphere. An intra molecular S(6) ring motif is formed by N3 hydrogens with the halide ions in all the crystal structures.

### Crystal structure description of [FAMNCL] 1 & Crystal structure description of [FACOCLI] 2

The asymmetric unit of **(1)** as well as **(2)** consists of two metal centers with similar distorted octahedral coordination geometry (made up of two furfuryladenine cations, three chloride ions and a water molecule). A lattice chloride ion lies at general position and two lattice chloride ions are present in special position (each with 0.5 occupancy).

**Figure 1:**
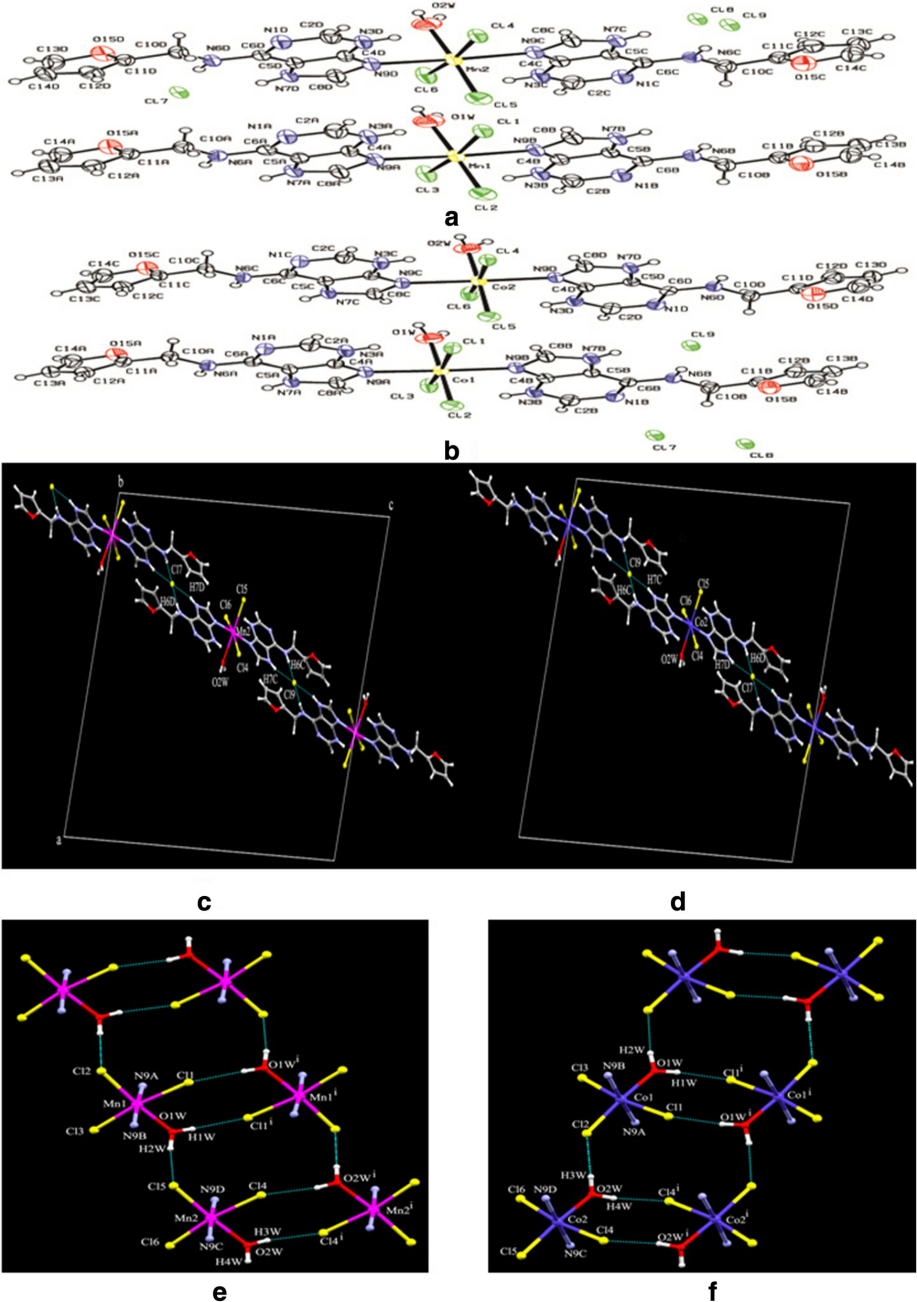
**ORTEP views of** ***1 & 2.*** **(a, b)** ORTEP views of 1 & 2respectively. **(b, c)** Chain formed by the Hoogsteen site hydrogens and lattice chloride ions in *1 & 2* respectively. **(c, d)** Supramolecular ladder formed by the coordinated water and chloride ions via O-H…Cl interactions in 1 and 2 respectively.

The special position chloride ions (Cl7 and Cl8 for complex **(3)** where as Cl8 and Cl9 for complex **(3)**) are positioned at e 2 Wyckoff site symmetry [34-36]. The Hoogsteen site hydrogens are involved in N-H…Cl hydrogen bonds with the lattice chloride ion which leads to a chain like pattern in the crystal structures **(1)** and **(2)** respectively (Figure 1c,d). Crystal structure **(1)** contains cobalt ion and **(2)** contains manganese ion as metal centers, but all other groups are similar in the asymmetric unit. The inter ligand interactions involving the chloride ions and the water molecules generate a ladder like pattern (Figure 1c,d). In the ladder M1 and M2 metal centers (Mn1and Mn2 for **(1)** and Co1 and Co2 for **(2)**) are arranged alternatively (Figure 1e,f).

**Figure 2:**
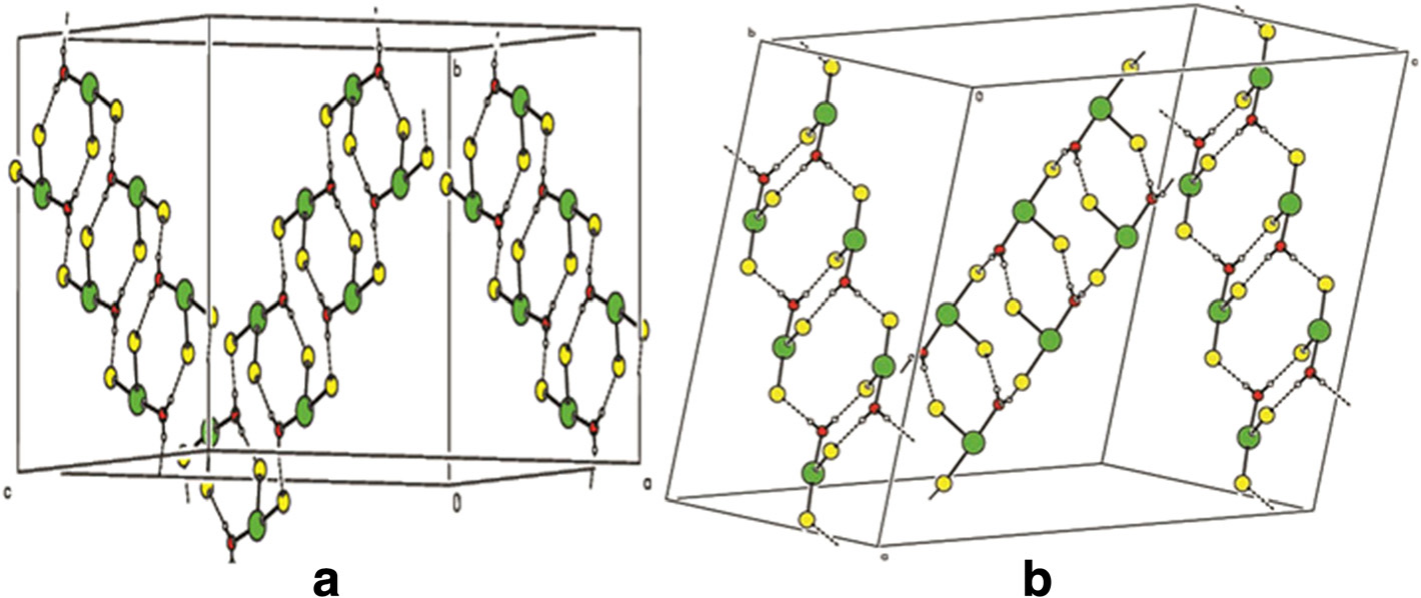
**The scissoring arrangement of supramolecular ladders. a, b)** The scissoring arrangement of supramolecular ladders in 1 and 2 respectively.

In these supramolecular ladders the coordinated water and coordinated chloride ions in the structures **(1)** and **(2)** form R_4_^4^(12) and R_2_^2^ (8) ring motifs made up of O-H…Cl interactions. Perpendicular to the R_2_^2^(8) motif, R_3_^2^(9) motif is made by the same chloride and water with adenine ring N3 & C2 hydrogens. The ladders are arranged in a scissoring arrangement in the packing pattern of the crystal structures **(1)** and **(2)** respectively (Figure 2a,b). The supramolecular chains are connected by supramolecular ladders and this leads to a three dimensional network which is also further stabilized by the C10-H…Cl interactions in both **(1, 2)**.

### Crystal structure description of [FACOCLII] 3

**Figure 3:**
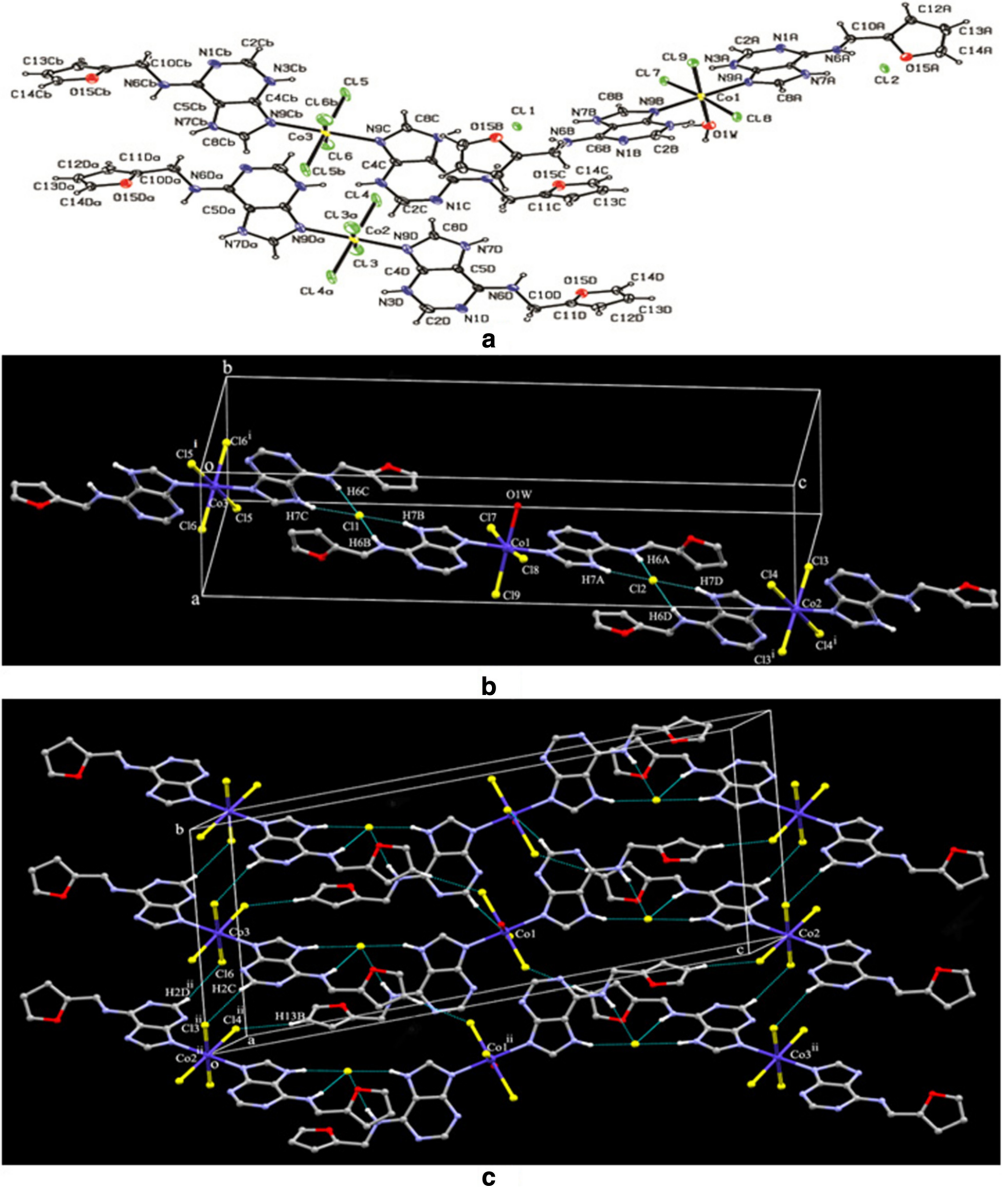
**ORTEP view of (3). a)** An ORTEP view of (3) showing the atom labelling scheme. Displacement ellipsoids are drawn at 20% probability level. **b)** Chain observed in FACOCLII. Hydrogens not involved in hydrogen bonding are omitted for clarity. **c)** Supramolecular ladders interlinked by a set of N-H…Cl interactions at the Hoogsteen site.

The asymmetric unit consists of three mononuclear cobalt centers and two lattice chloride ions. All the three Co centers have a distorted octahedral geometry. Two of the cobalt centers (each on an inversion center) have similar coordination geometry made up of two chloride ions, one furfuryladenine cation and their inversion related ligands (Figure 3a). They are (Co2 and Co3 ions) positioned at *a* ī and *e* ī Wyckoff site symmetries respectively. Unlike these Co2 and Co3 ions, Co1 ion lies at the general position (surrounded by the two furfuryladenine cations, three chloride ions and a hydronium ion). This cobalt center (Co1) also forms R_4_^4^(12), R_2_^2^(8), R_3_^2^(9) and the ladder type arrangement, which is similar to that of crystals **(1)** and **(2)**, which is the unique character observed in Co1 and not in Co2 or Co3 centre. A supramolecular chain is formed by the Hoogsteen site hydrogens and a lattice chloride ion. In this chain the cobalt centers are arranged as (-Co2-Co1-Co3-Co1-Co2-Co1-Co3-)_n_ revealing the alternative arrangement of Co1 inbetween (Co2 and Co3) as well as (Co3 and Co2) (Figure 3b). Supramolecular ladders are interlinked by a set of N-H…Cl interactions at the Hoogsteen site. (Fig3.3c).

### Crystal structure description of (FANICL) 4

**Figure 4:**
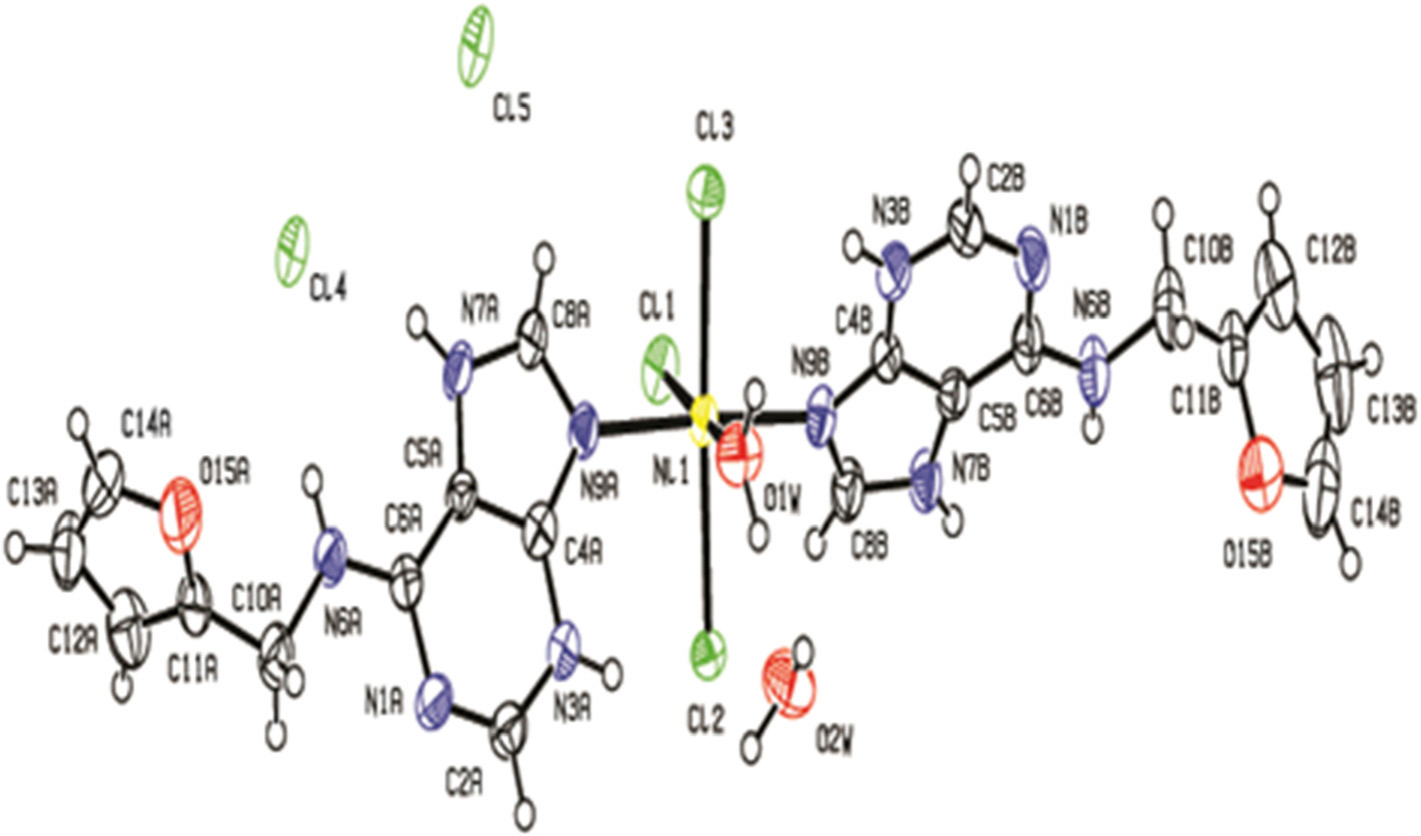
**An ORTEP view of (4) showing the atom labelling scheme.** Displacement ellipsoids are drawn at 50% probability level.

The asymmetric unit consists of a cobalt ion with distorted octahedral coordination geometry, coordinated to two furfuryladenine cations and two chloride ions equatorially, a chloride ion and a water molecule axially (Figure 4).

**Figure 5:**
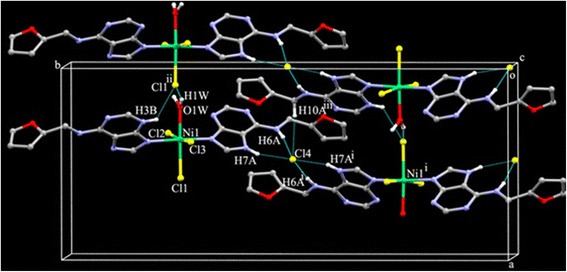
**The metal centers connected by a O-H…O,C-H..Cl and two O-H…Cl interactions as well as Chain formed by N-H…Cl interactions in between the Hoogsteen site hydrogen and lattice chloride ion**.

In the lattice, two chloride ions with 0.5 occupancy and a water molecule are present. The N-H…Cl interactions found in between the Hoogsteen site hydrogen and lattice chloride ion generates a chain which is also observed in **(1-3)** (Figure 5). Two of the metal centers are connected by a O-H…O,C-H..Cl and two O-H…Cl interactions inbetween a coordinated water molecule, lattice water molecule and two of the coordinated chloride ions (Figure 5). One of the lattice water hydrogen is involved in bifurcated hydrogen bond with two coordinated chloride ions forming a R_1_^2^(4) motif. The C-H…Cl interaction is present inbetween the C2 hydrogen of the coordinated furfuryl adenine and the chloride of the next cobalt centre. O-H…O, two C-H…Cl, two O-H…Cl and N-H…Cl interactions generates a three dimensional network by connecting two of these chains (Figure 5).

### Crystal structure description of [Zn(BAH+)Br3] (BAZNBR) 5

**Figure 6:**
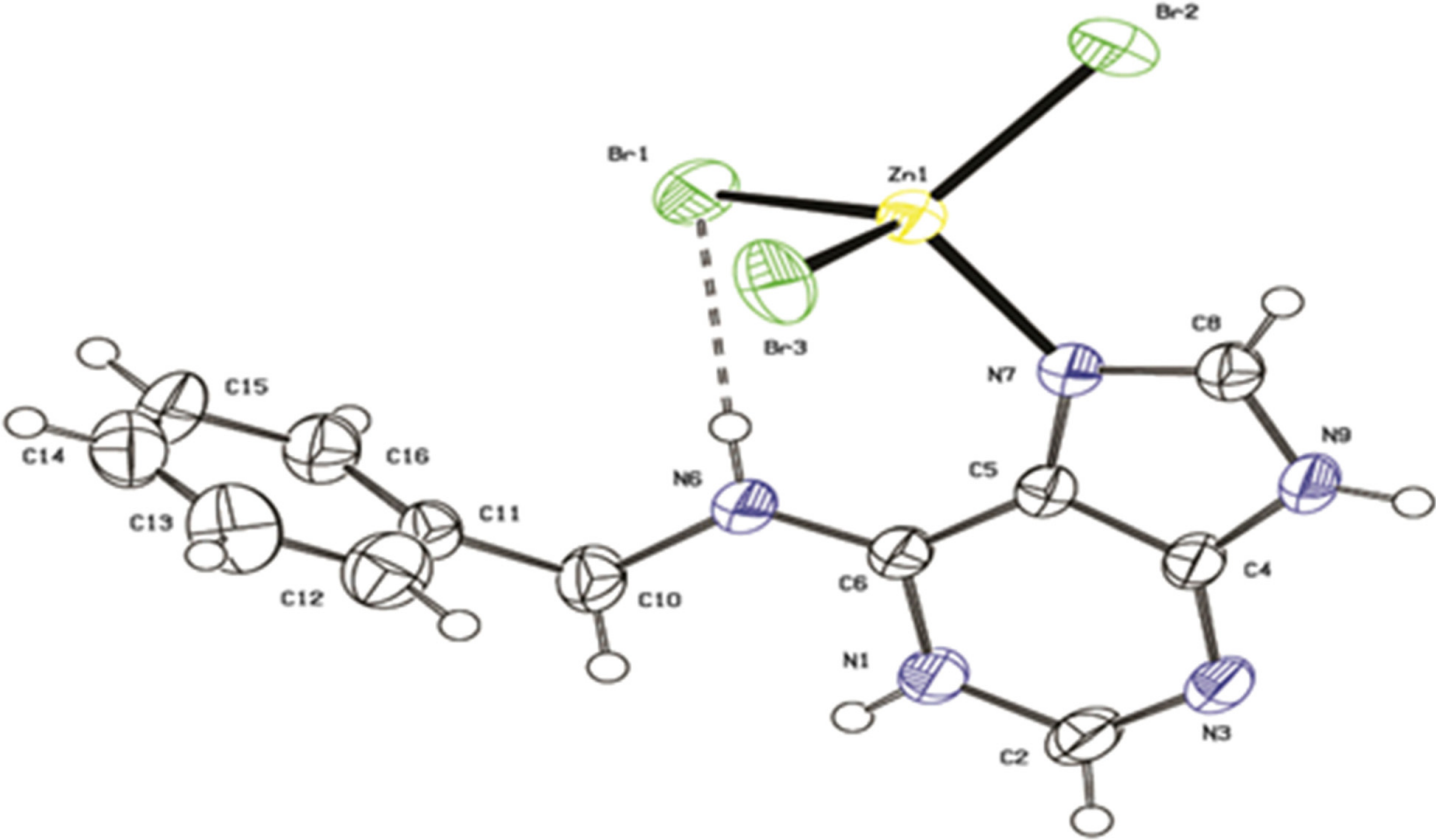
**An ORTEP view of (5) showing the atom labelling scheme.** Displacement ellipsoids are drawn at 50% probability level.

The asymmetric unit consists of a zinc(II) metal center, terahedrally coordinated to benzyladeninium cation (BAH^+^) and three bromide ions. The BAH^+^ is a N7-coordinated; N1 protonated; N9-H tautomer (Figure 6).

**Figure 7:**
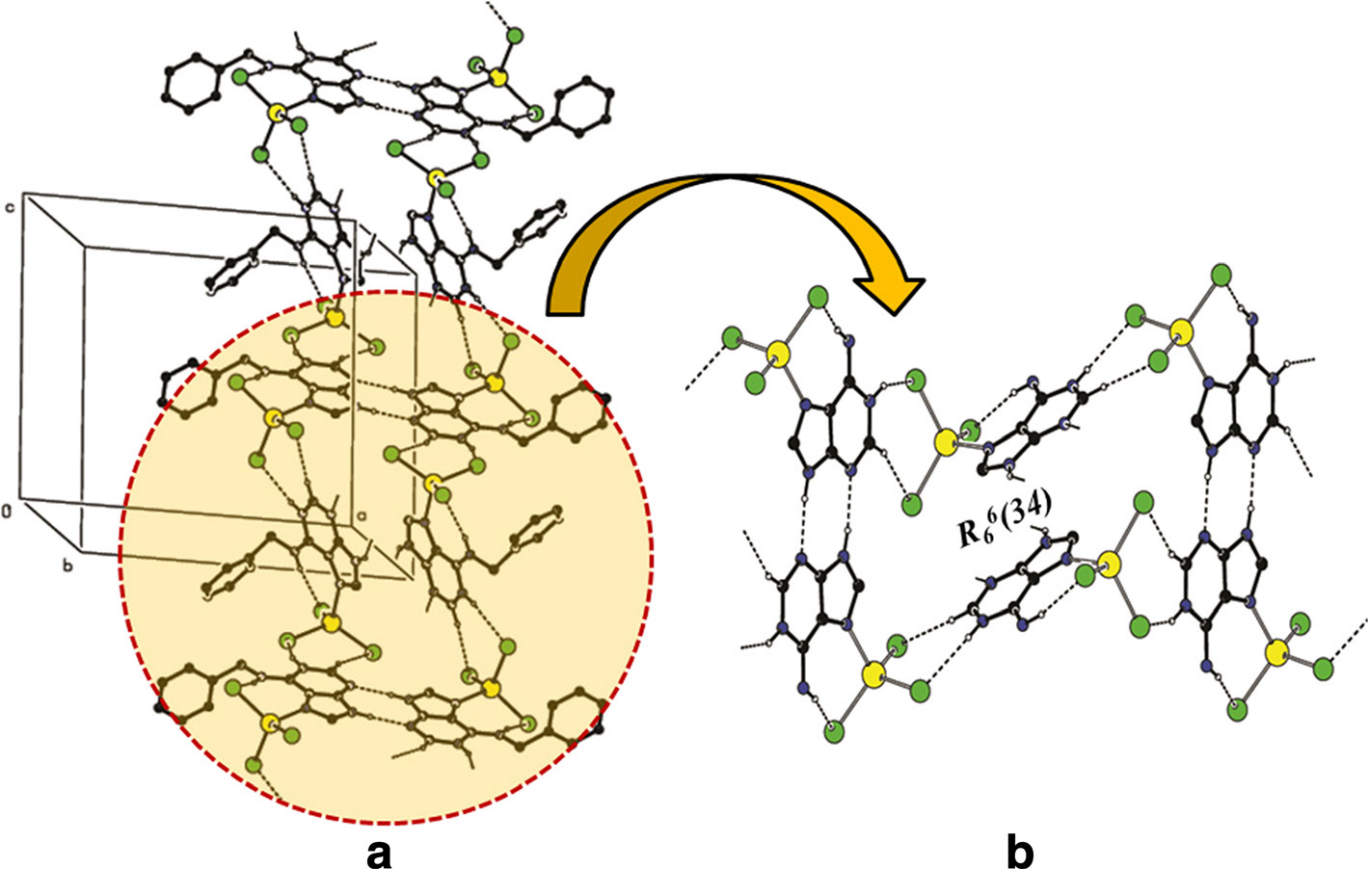
**The formation of N-H…N base pair. a)** The formation of N-H…N base pair between alternative adenine moieties which lie in same plane. **b)** R_6_^6^(34) ring motif is formed between two chains through the N-H… Br and C-H… Br bonds.

The Br1 interchelated by the N6-H atom through N-H…Br hydrogen bond forms a S(7) motif. The N1 and C2 atoms form hydrogen bonds with two different bromide ions leading to a R_2_^2^(7) ring motif. The continuous formation of R_2_^2^(7) ring motif extends a chain along c axis (Figure 7a). In this chain adjacent adenine moieties are in different planes and forming a N-H…N base pair with two different chain adenine moieties, but alternate adenine moieties; are in same plane and form a base pair with the same chain adenine moieties (Figure 7a).

**Figure 8:**
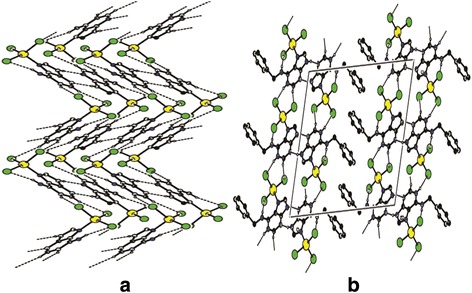
**The base pair connecting the successive chains. a)** The base pair connecting the successive chains leading to a three dimensional network. The benzyl substituents are omitted for clarity. **b)** The stacking between the phenyl rings of the adenine moieties along b axis.

R_6_^6^(34) ring motif is formed between these two chains through the N-H… Br and C-H… Br bonds (Figure 7b). A single chain is connected through base pair with two different chains. These two chains are further connected to different chains and so on, generating three dimensional networks. (Figure 8a). The phenyl rings of symmetry related molecules are stacked with one another giving additional stabilization to the structure (Figure 8b).

### Crystal structure description of (BACDBR) 6

**Figure 9:**
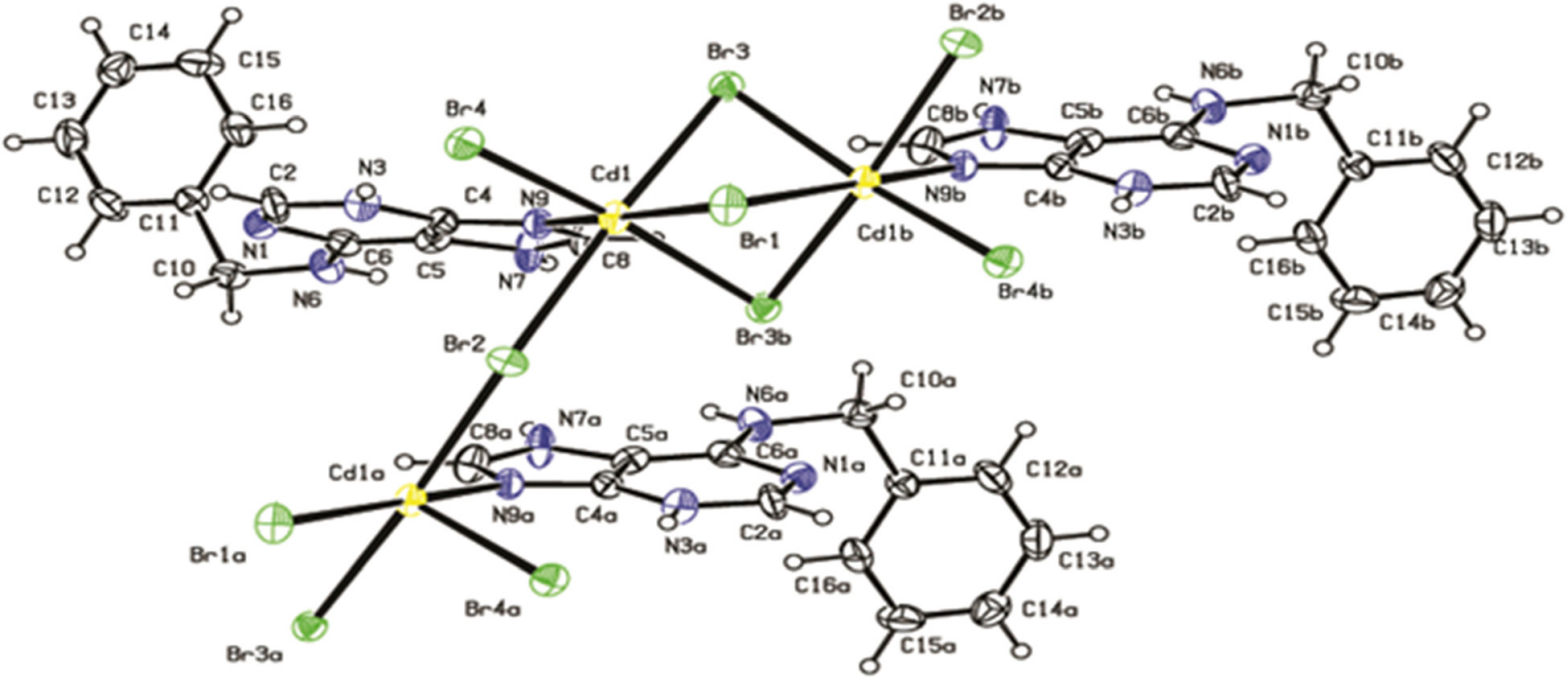
**An ORTEP view of (8) showing the atom labelling scheme**. Displacement ellipsoids are drawn at 50% probability level.

**Figure 10:**
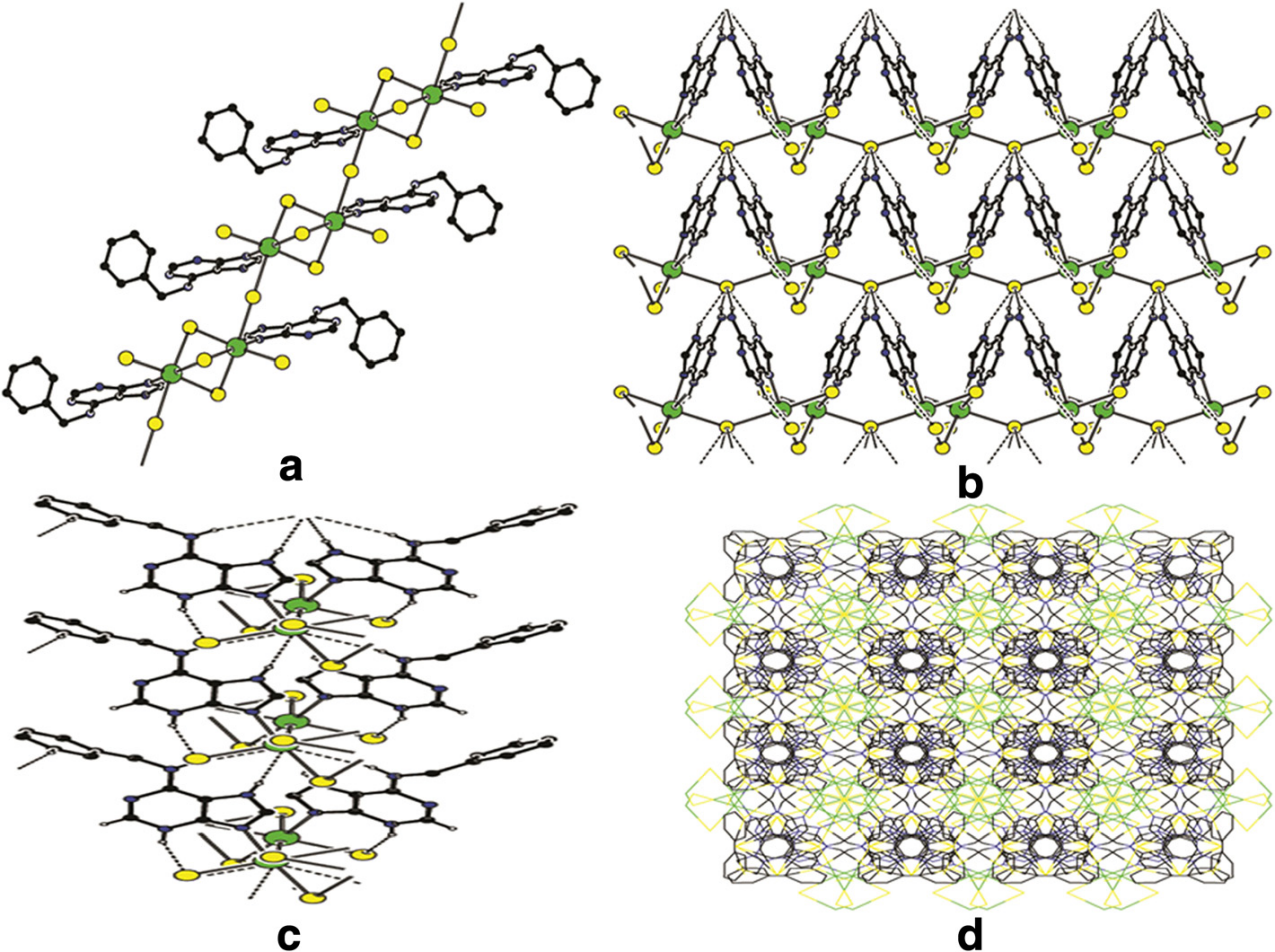
**Coordination polymer formed by the monobridged bromide ion. a)** Coordination polymer formed by the monobridged bromide ion (Br_2_) linking the dicadmium center. **b)** Coordination polymeric chains linked by the Hoogsteen site hydrogens and monobridged Br_2_ ions via N-H…Br hydrogen bonds. **c)** C-H…π interaction between C2 hydrogen and the phenyl ring of BAH+. **d)** Formation of three dimensional networks viewed along c axis.

The crystal structure of **(6)** is a coordination polymer and it consists of two cadmium centers bridged by three bromide ions. Each of the cadmium center is six coordinated by five bromide and one benzyadeninium cation (BAH^+^) (Figure 9). Each of the dicadmium centers are linked to each other by a bromide ion and this extends the structure into a coordination polymer (Figure 10a).

The cadmium ions in this dicadmium center are separated by the distance of 3.467 Å. In this polymeric chain, all BAH^+^ ions are arranged in one side. The chains are connected by the Br2 bromide ions and the Hoogsteen site hydrogens *via* N-H..Br hydrogen bonds (Figure 10b). Polymeric chain interlinks generate “V” shaped arrangement repeatedly in the network. Each cadmium has one bromide ion, which is not involved in bridging, which forms a S(6) intramolecular ring motif with the N3 hydrogen of BAH^+^. C-H…Br interaction is found between C15 hydrogen of the phenyl ring and the Br4. A weak C-H…π interaction is observed between C2 hydrogen and phenyl ring of the BAH^+^ (Figure 10c). Three dimensional network of the crystal structure viewed along c axis is shown in (Figure 10d).

## Conclusion

We have obtained a series of metal complexes of N6-furfuryl adenine/N6-benzyl adenine. Of these six, five are mononuclear while the one is a coordination polymer. Complexes show two different coordination geometries like distorted octahedral in **1-4, 6** and tetrahedral in **5)**. In complexes **(1-4)** the primary supramolecular organization remain common which is a chain made up of N-H…Cl interactions inbetween the Hoogsteen sites hydrogen and a Chloride anion lying at the lattice. These crystal structures show several supramolecular motifs, stacking patterns, preferred site of protonation/coordination, etc., in these kinds of complexes. This piece of work may also help in study of similar type complexes which may eventually lead to novel functional materials.

### Supplementary material

CCDC 1005990-1005993, 1005996 and 1005997 contain the supplementary crystallographic data for structures **(1-6)** respectively can be obtained free of charge via http://www.ccdc.cam.ac.uk/Community/Requestastructure/Pages/DataRequest.aspx?, or from the Cambridge Crystallographic Data Center, 12 Union Road, Cambridge CB2 IEZ, UK; fax:(+44)1223-336-033; or e-mail: deposit@ccdc.cam.ac.uk.
